# Measurement and Modeling of Short and Medium Range Order in Amorphous Ta_2_O_5_ Thin Films

**DOI:** 10.1038/srep32170

**Published:** 2016-08-26

**Authors:** Badri Shyam, Kevin H. Stone, Riccardo Bassiri, Martin M. Fejer, Michael F. Toney, Apurva Mehta

**Affiliations:** 1 Stanford Synchrotron Radiation Lightsource, SLAC National Accelerator Laboratory, Menlo Park CA 94025, United States; 2 E. L. Ginzton Laboratory, Stanford University, Stanford CA 94305, United States

## Abstract

Amorphous films and coatings are rapidly growing in importance. Yet, there is a dearth of high-quality structural data on sub-micron films. Not understanding how these materials assemble at atomic scale limits fundamental insights needed to improve their performance. Here, we use grazing-incidence x-ray total scattering measurements to examine the atomic structure of the top 50–100 nm of Ta_2_O_5_ films; mirror coatings that show high promise to significantly improve the sensitivity of the next generation of gravitational-wave detectors. Our measurements show noticeable changes well into medium range, not only between crystalline and amorphous, but also between as-deposited, annealed and doped amorphous films. It is a further challenge to quickly translate the structural information into insights into mechanisms of packing and disorder. Here, we illustrate a modeling approach that allows translation of observed structural features to a physically intuitive packing of a primary structural unit based on a kinked Ta-O-Ta backbone. Our modeling illustrates how Ta-O-Ta units link to form longer 1D chains and even 2D ribbons, and how doping and annealing influences formation of 2D order. We also find that all the amorphousTa_2_O_5_ films studied in here are not just poorly crystalline but appear to lack true 3D order.

Understanding the relationship between material properties, chemistry and atomic scale structural arrangement is critical for discovery and optimization of new functional materials. The structural arrangement of nearest neighbors around any atom, dictated largely by bond strength and atomic size, encapsulates the “chemical order” and is fairly robust to synthesis conditions and other external drivers. However, going further away from the short-range order (SRO), small changes (e.g., in dihedral angle) arising from entropy or weaker but longer-range forces makes the atomic arrangement less robust and more prone to change with changes in processing conditions and other external influences. These changes are reflected in the arrangement of atoms in 0.5–5 nm range, commonly referred to as medium-range order (MRO) and many desirable properties ranging from magnetism, electronic properties to mechanical stiffness emerge from these longer-range interactions^1^. In the extreme limit of fully disordered materials when long-range symmetry disappears, the MRO remains arguably our most valuable source of structural information^2^.

Sub-micron amorphous films are of growing scientific and technological importance. For instance, improvements in the amorphous mirror coatings have played a critical role in the first detection of gravitational waves^3^. They also find widespread use as high-k gate oxides^4^, transparent conducting oxides^5^, corrosion resistance coatings^6^, and advanced phase-change memory^7^. Amorphous films also allow greater control of composition and formation of metastable materials. Properties such as refractive index^8^, conductance/resistivity^9^, magnetic susceptibility^10^, hardness^11^ and the glass transition temperature^12^ of amorphous films are known to change significantly with thickness. Thus, amorphous materials can be induced to exhibit a wider range of properties in thin film form, which are difficult or even impossible to achieve in bulk^13^. Obtaining structural insight into the MRO in amorphous films, therefore, offers a tremendous opportunity towards developing a comprehensive understanding of structure-property relationships in disordered materials.

Pair Distribution Function (PDF) analysis of total elastic scattering (Bragg and diffuse) is perhaps the most powerful method to access the MRO. Neutron, electron and x-ray scattering have long been used to study disordered materials including liquids, glasses and amorphous materials^14^,^15^. Despite the power of these techniques, it is extremely challenging to obtain high quality PDF from sub-micron thick amorphous films due to low scattering volume and contamination of the total scattering signal from the substrate. The high penetration and low brightness of neutron sources makes it impossible to obtain scattering data from amorphous films in a reasonable amount of time. Electron PDF studies require very thin cross-sections (typically below 20 nm) leading to the possibility of altered cross-sections (due to thinning processes) and/or loss of templating influence of the substrate. Further, even though limitation due to small sample volumes and corrections due to multiple scattering effects have been largely overcome^16^,^17^, until recently it has been difficult to collect electron-PDF data on amorphous films to momentum transfer values (Q) beyond 12 Å^−1^. X-ray total scattering measurements have the potential to overcome most of these challenges but require very intense, small and highly collimated, high-energy beams. Many x-ray PDF measurements on sub-micron films eliminated the problem of substrate scattering through use of ‘delaminated’ films^18^,^19^. However, both electron PDF, and PDF from delaminated films are destructive measurements. Recently, there was a report of a non-destructive tour-the-force measurement by Jensen and co-workers^20^ in which they isolate the total scattering of a 360 nm film grown on a relatively thin (170 microns) borosilicate glass by careful subtraction of the substrate contribution. Obtaining total scattering from a thin film via carefully subtraction of scattering from the substrate, despite being non-destructive, has some inherent limitations. The measurements are limited to relatively thin and poorly scattering, low-Z substrates. Further difficulties arise if there is a thin “sticking/wetting” layer between the substrate and the thin film, as is the case for many functional thin films. Furthermore, many amorphous films are formed by rapid cooling (e.g. melt spun ribbons) or layer-by-layer growth (e.g. atomic layer deposition) and may have structural gradients across the thickness. Transmission measurements would average over structural variations across the thickness of the film. Almost 30 years ago Fischer-Colbrie, Bienenstock and co-workers^21^,^22^ obtained total scattering measurements from amorphous films by carefully controlling the incidence angle. There are a few other recent studies grazing-incidence pair distribution measurement, but all of these measurements go no higher than a Q_max_ of 11 Å^−1^. Limited Q range (of 11 Å^−1^ or less), results in lower resolution (of 0.5 Å) pair distribution masking many subtle but important structural shifts and distortions. Thus, even after 30 years of work, there is still a notable scarcity of high-quality x-ray PDF data from amorphous films.

In this work, we demonstrate that it is possible to obtain very high-quality total x-ray scattering measurements (with Q range of 20 Å^−1^) from the top 50–100 nm of 500 nm amorphous Ta_2_O_5_ films in grazing-incidence geometry. Our approach is non-destructive and can measure films grown on any flat substrate. By carefully controlling the incidence angle it can probe variation in the structure as a function of depth. It can, thus, easily handle a sticking/wetting layer and also probe very thin amorphous films (~10 nm or less). Our approach has many advantages over the approaches cited above and is ideally suited to probe subtle changes in the short and medium range-order in a growing number of scientifically and technologically important sub-micron amorphous films and coatings.

A common means of understanding the structure of amorphous materials is currently based on modeling of the PDF (e.g. the Reverse Monte Carlo method)^23^ often with optimization from energy minimization routines such as Molecular Dynamics^24^. Though powerful, these approaches output clusters of several hundred atoms and do not separate structural building blocks from disordered atoms. Without further modeling or complementary information from other techniques, they do not give any direct physical insight into how a structure is put together and the role disorder plays in disrupting it.

In here, we also demonstrate a physically intuitive approach for extracting short and medium-range atomic arrangement from the PDF of amorphous materials. Our approach is based on the concept that local (i.e., short-range) arrangement of atoms in materials is dictated by chemistry and bonding constraints and is an intrinsic characteristic of a compound. In most materials, these Primary Structural Units (PSUs) or motifs, though derived from chemistry, are topological entities that are not identical to (or may be different from) the fundamental chemical unit. Modeling of MRO based on the topological packing of PSUs leads to quick insights into possible frustrations and disorder that hinders emergence of long-range order. We use this approach to gain insight into effects of doping, and annealing of tantala thin films.

The goal of this paper is therefore two-fold: first to show that with sufficient care, high-quality grazing-incidence pair distribution function (GIPDF) data may be obtained from sub-micron amorphous films. Second, taking amorphous tantala as an example, to present an intuitive modeling approach that provides a counterpoint to computationally intensive approaches based on Molecular Dynamics and Reverse Monte Carlo methods to obtain a fundamental understanding of the MRO in amorphous and glassy films.

## Results and Discussion

**Figure 1:**
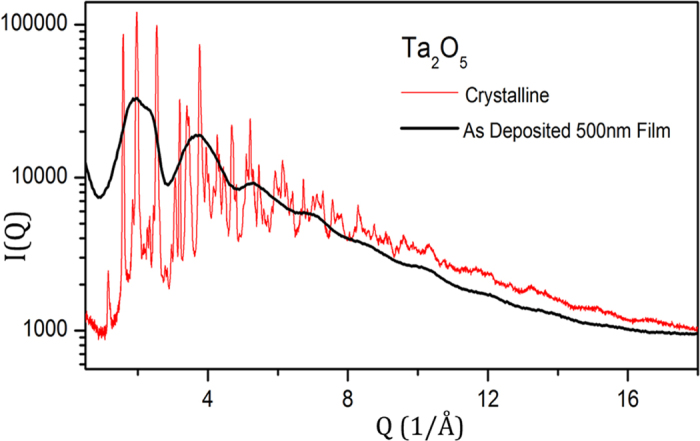
X-ray total scattering data for crystalline (in red) and an amorphous Ta_2_O_5_ thin film (in black). The scattering data from the amorphous film was multiplied by a scale factor to facilitate comparison of the two data sets.

**Figure 2:**
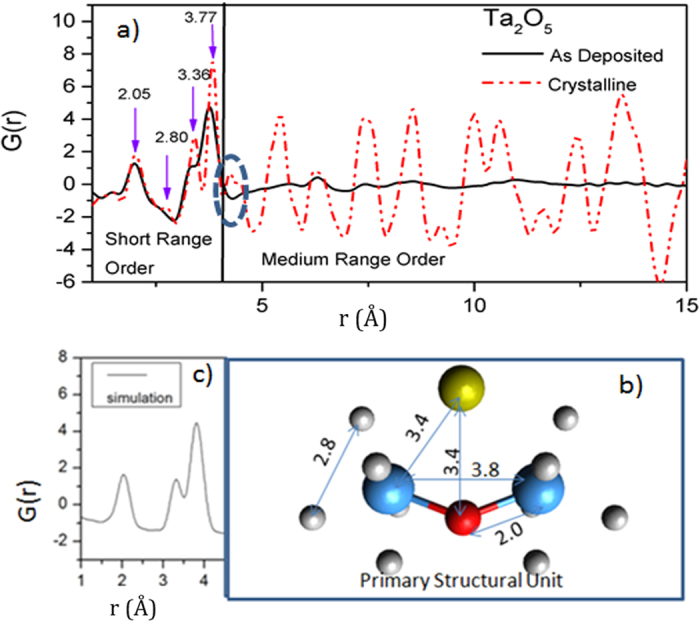
(**a**) Experimental pair distribution function data for crystalline (dashed red line) and amorphous Ta_2_O_5_ (solid black line); (**b**) The derived primary structural unit (PSU); the link shown by blue and red spheres is the Ta-O-Ta backbone, the gold sphere shows the metal interstitial and the grey sphere (disordered) oxygens; and (**c**) the calculated theoretical pair distribution function for the PSU.

The elastic x-ray scattering data, I(Q), for crystalline a Ta_2_O_5_ powder and an as-deposited amorphous Ta_2_O_5_ film are shown in Fig. 1. As expected, the crystalline Ta_2_O_5_ (tantala) shows numerous sharp diffraction peaks whereas the as-deposited tantala film displays smoothly varying, broad features characteristic of an amorphous material. Yet note that the diffuse scattering intensity in the two samples is not dissimilar. The total scattering data, I(Q), were corrected for air scattering, absorption, Lorentz and polarization effects, Compton scattering and geometric effects associated with detector footprint. The corrected data were normalized to the composition to obtain the x-ray weighted total structure factor, S(Q). The S(Q) data at higher Q was smoothly damped to zero using a Lorch function^25^ to minimize Fourier truncation artifacts. The resulting real-space pair distribution functions (PDF or G(r)) are shown in Fig. 2a. (It is worth pointing out that to observe the separation of the 3.4 Å peak from 3.8 Å peak total scattering data up to Q_max_ of 15 Å^−1^or greater is necessary).

If one compares the PDF data for the crystalline and amorphous tantala, shown in Fig. 2a, they are strikingly similar below 4 Å, but very quickly diverge at longer distances. The close similarity in the short-range order (below 4 Å) allows us to invoke the idea that both materials are constructed from almost identical building blocks. We will call them primary structural units (PSUs) and the two materials mostly differ in how these PSUs are arranged in medium and long range. This idea of thinking of materials as packing and ordering of PSUs is an old but powerful concept, widely used in crystallography for deconstructing large and complex structures into smaller blocks. Lately, it is most commonly found in macromolecular crystallography where a complex protein is constructed from primary structural units such as alpha helices and beta sheets^26^. The novelty here lies in its application to describe the medium range structure of an amorphous material. The PSU we will use is not the fundamental chemical unit or a rigid body, but just the essential topological skeleton; it is often decorated with additional atoms and distorts in response to internal and external forces.

The Ta ions in these materials are known to reside in the center of corner- and edge-shared polyhedra^27^,^28^,^29^. Recently published Ta-centered local structure from Ta L_3_ edge EXAFS data^30^,^31^ indicate that the Ta ions are surrounded by between 5 and 6 oxygens. There are different ways in which these Ta centered polyhedra connect to each other to give rise to different polymorphs. We find that, though the Ta-centered polyhedra are essential for understanding very local “chemical order”, they are not the primary units for understanding the topological packing and order in tantala. Extracting the bond distances and average coordination number constrains (shown in Fig. 2b), from Ta-centered information from Ta L_3_ edge EXAFS data^30^,^31^ and O-centered information from NMR spectroscopy^32^, we construct the backbone of the topological PSU as a kinked Ta-O-Ta chain, with a Ta-O bond of ca. 2.05 Å and a Ta-O-Ta angle of approximately 135–150 degrees. This backbone is decorated by (two) metal ‘interstitial’ site(s) (one shown at the top of Fig. 2b by a gold sphere). The interstitial sites are often only partially occupied, but when occupied the coordination of the backbone oxygen increases from 2 to 3. The PSU with one of the interstitial site occupied is reminiscent of the structure motif in Anatase^33^ and Zirconia^34^, albeit significantly distorted, and suggest a possible substitution of Ti or Zr into tantala. The two Ta atoms of the backbone are decorated by polyhedra of oxygens, shown in Fig. 2b as small grey spheres, necessary to complete the fundamental chemical unit and charge balance in tantala. The small peak at 2.8 Å corresponds to the adjacent O-O distance from these chemical units. Suppression of this peak in the amorphous films indicates that the oxygen polyhedra are considerably disordered.

The PSU described above accounts for all of the short-range correlations seen in both crystalline and amorphous tantala (as shown by the correlations marked on Fig. 2b). The higher Q-range of our data allows resolution of pair correlations that are not fully resolved in the earlier studies of amorphous tantala (for example, the 3.4 Å from the 3.8 Å peak). Figure 2c shows a simulation of the PDF based on the PSU proposed above, calculated using the PDFgui software package^35^, indicating again that the PSU adequately represents the short-range structure of amorphous tantala. The assignment of pair correlations below 4 Å are broadly consistent with recent studies on amorphous tantala as well as tantala-based glasses^36^,^37^,^38^,^39^,^40^. Taken together, the kinked Ta-O-Ta backbone of the proposed PSU appears to be a common, but previously unrecognized, fundamental building-block. It is also visible as a prominent motif in DFT calculations^41^, Reverse Monte Carlo simulations of the electron-PDF data^42^, high temperature orthorhombic structure^27^, as well as in published structures of the δ and β polymorphs of tantala^28^,^29^. (See the figure in the supplementary material, which shows the proposed kinked Ta-O-Ta PSU backbone highlighted in δ and β polymorphs of tantala.)

**Figure 3:**
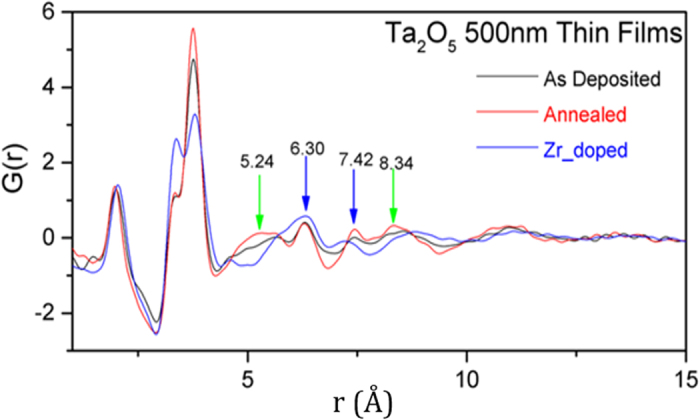
Pair distribution functions for pristine (as deposited, room temperature), annealed (600 °C) and doped (Zr) Ta_2_O_5_ thin films, shown as black, red and blue curves respectively.

Figure 3a shows a comparison of the PDF of the as-deposited film with a film annealed at 600 °C and a film doped with 35% Zirconia (Zr-Ta_2_O_5_). The first observation is that PDF of all three amorphous films show at least some order up to 1 nm. This is in agreement with recent fluctuation electron microscopy (FEM) and electron-PDF studies where evidence for ordering up to 1–1.5 nm was found in amorphous Ta_2_O_5_ and Ti-doped Ta_2_O_5_ thin films^27^,^43^. The second observation is that even though the overall PDF looks similar for all three films, there are significant differences among them, not only in the short-range correlation but also in medium-range order. There are subtle differences in the first metal-oxygen correlation: the average metal-oxygen bond distance contracts slightly on annealing, and expands slightly on Zr doping. The most prominent short-range changes, however, are seen in the amplitude of the metal-metal (second neighbor) correlations, especially on Zr doping, suggesting that more interstitial sites are occupied in Zr-doped sample; perhaps not surprising, considering the similarity of the interstitially occupied tantala PSU to the short-range structure of zirconia.

**Figure 4:**
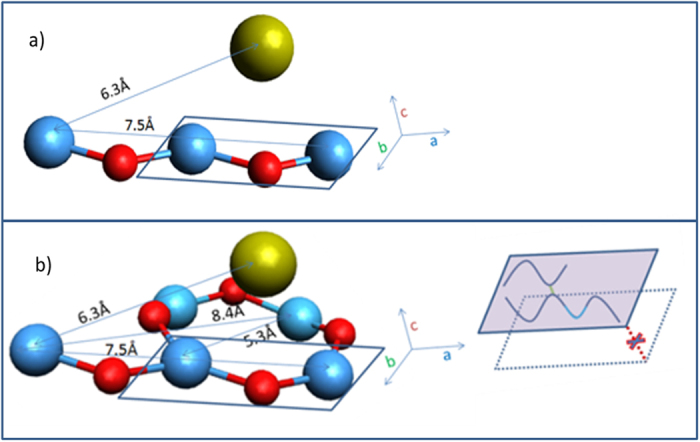
A schematic linking of PSUs for amorphous Ta_2_O_5_ that account for the pair correlations seen beyond 4 Å (in Fig. 3). Single PSU is outlined by the blue rectangle; (**a**) Two PSUs linking to form a zigzag chain; and (**b**) and a zigzag chain cross-linking with a PSU to form a ribbon. Inset in (**b**) schematically depicts formation of 1D (chains) and 2D (ribbons) order, but lack of 3D ordering. (Oxygens not necessary for the topological order, but needed for chemical balance-the light grey spheres in Fig. 2- are not shown here).

More intriguing differences among the PDFs of the three amorphous films are, however, in the MRO. There are four salient correlation features that capture these differences, viz. at 5.24 Å, 6.30 Å, 7.42 Å and 8.34 Å respectively, and are most prominent in the 600 °C annealed thin film. The 6.3 Å and 7.4 Å distances, we believe, correspond to the M-M distance (and O-O distance) when two PSUs link end-to-end, forming a longer zigzag chain, as shown in Fig. 4a. The 6.3 Å (and perhaps even the 7.4 Å) correlation is prominent in all the films studied here, suggesting the prevalence of short Ta-O-Ta zigzag chains in them.

The other two correlations, i.e., at 5.3 and 8.3 Å, correspond to cross-linking of two PSUs to form a ribbon, as depicted in Fig. 4b. These features are most revealing of the effect of annealing or Zr doping. They, especially the 5.3 Å correlations, become sharper and more pronounced upon annealing, indicating that annealing increases the amount of the cross-linking of PSU chains to form short ribbon fragments. However, the 5.3 Å feature seems to disappear upon Zr doping, suggesting that dominant influence of Zr doping on the MRO in amorphous tantala is to alter and perhaps suppress formation of ribbons (i.e., 2D order).

Another way of viewing crosslinking of Ta-O-Ta chains is to think of formation of Ta-O-Ta rings. Our model of amorphous tantala suggests that rings smaller than those composed of 4 Ta and 4 O atoms are less likely to exist in amorphous tantala. (Fig. 4b) Any closed rings made of 2 PSUs (i.e. 2Ta and 2O) would place the oxygens too close to each other, and no such short correlations are visible in the PDF. Three PSU rings (i.e. 3Ta and 3O) would suppress the 5.3 Å and 8.4 Å correlations and generate a new correlation at 5 Å. The PDF of the Zr-doped data does show suppression of the 5.3 Å and 8.4 Å correlations, but does not show any intensity at 5 Å, suggesting a very low likelihood of finding closed rings comprising 2 or 3 PSUs in amorphous tantala.

Equally important to an understanding of the MRO of amorphous tantala are the missing pair correlations near 4 Å, clearly visible in the MRO of crystalline tantala (highlighted by the dotted oval in Fig. 2a), which are signatures for packing perpendicular to both the chain and the chain cross-link direction (c axis in Fig. 4b). The absence of these correlation peaks, even in the 600 °C annealed sample, indicate that these films are not just poorly crystalline, but appear to lack true 3D order. All of the other pair correlations discussed above are also present in the crystalline structure; however, the ones in the amorphous films are about an order of magnitude weaker, suggesting that, though the MRO in the amorphous films may lack 3D order, it is otherwise similar to crystalline MRO, but about an order of magnitude weaker in the 5–10 Å range.

## Conclusions

In conclusion, we report high quality grazing-incidence x-ray total scattering data from the top ~50–100 nm of 500 nm (doped and annealed) amorphous tantala thin films. These data are among the highest quality total scattering data reported for any sub-micron amorphous thin film to date. Though recent studies using various experimental techniques including EXAFS, NMR, electron-PDF and FEM have each revealed some key characteristics of the atomic structure of amorphous tantala, we believe that the structural insight obtained in this study, viz. the extraction of the kinked Ta-O-Ta structural motif and an understanding of how they link together, has revealed the most complete description of the short and medium-range atomic arrangements in amorphous tantala to date. Two salient structural features emerge from our analysis. First is that amorphous tantala even when annealed to 600 °C appears to lack true three-dimensional order, but does contain well-defined structural entities, some of which appear to be 1 nm or longer. These structural entities are short chains or 2D ribbons comprised of a zigzagging Ta-O-Ta backbone. The second insight to emerge from our analysis is that annealing increases the degree of cross-linking, whereas doping with Zr suppresses it. Annealing and doping both have significant effects on mechanical loss, a property critical for mirror coatings for Gravitational Wave detectors, suggesting that the emergence or suppression of medium range 2D order may have an important influence on properties of amorphous tantala.

## Materials and Methods

Grazing-incidence total x-ray scattering measurements were carried out on high-quality, ion-beam sputtered tantala thin films (500 nm) deposited on SiO_2_ (MLD Technologies, Mountainview, CA). In addition to the as-deposited tantala films, one annealed film (600 °C for 24 h in air), one doped film (ca. 35 wt. % Zr) and crystalline Ta_2_O_5_ powder (Sigma Aldrich, annealed at 800 °C) were also studied. The scattering experiments were performed at beamline 10-2 of the Stanford Synchrotron Radiation Lightsource (SSRL) using a 21.5 keV monochromatic, focused x-ray beam of dimensions 0.05 mm by 0.40 mm. Before performing high-Q total scattering measurements, the samples were screened in grazing incidence x-ray diffraction measurements with a large area detector (but with lower incidence energy of 12.7 keV, and thus lower Q range). These quick screening measurements were used to ensure that the films were not inhomogeneous, nor anisotropic and sufficiently flat to be suitable for grazing-incidence total scattering measurements. The detector setup for high-Q total scattering measurements consisted of Soller slits and a silicon drift detector (Vortex™)^44^. A full multichannel analyzer (MCA) spectrum was collected at every scattering point and elastic, inelastic/Compton and fluorescence signals were extracted from spectrum analysis. Following careful alignment, x-ray reflectivity (XRR) data and a Ta L_α_ fluorescence signal as a function of incidence angle (angle curve) were collected for each sample. Based on the XRR and angle curve, the critical angle was determined and an incident angle was selected (ca. 0.13°-0.18°) to limit the penetration of the x-rays into the sample to less than 100 nm. Exceptional care was taken to ensure that the scattering data from the thin films were uncontaminated by the scattering from the silica glass substrate. The intensity of the first diffraction maxima from silica glass (1.44–1.55 Å^−1^)^45^,^46^ as a function of the incident angle was carefully monitored to confirm that the full total scattering pattern collected was indeed free from scattering from the substrate. Total scattering data was collected from momentum transfer (Q) of 0.1 to 20.5 Å^−1^, with a Q-weighted counting scheme to ensure that the data was of sufficiently high quality at least up to 20 Å^−1^. The resulting data quality is more than adequate for a detailed discussion of the local and intermediate structure, as seen in many earlier (x-ray transmission) studies on bulk amorphous materials^47^,^48^,^49^,^50^.

## Additional Information

**How to cite this article**: Shyam, B. *et al*. Measurement and Modeling of Short and Medium Range Order in Amorphous Ta_2_O_5_ Thin Films. *Sci. Rep.* **6**, 32170; doi: 10.1038/srep32170 (2016).

## Supplementary Material

Supplementary Information

## References

[b1] Fernandez M., Shi H. & Barnard A. S. Quantitative Structure−Property Relationship Modeling of Electronic Properties of Graphene Using Atomic Radial Distribution Function Scores. J. Chem. Inf. Model 55, 2500–2506 (2015). 26619798 10.1021/acs.jcim.5b00456

[b2] Price D. L., Moss S. C., Reijers R., Saboungi M. L. & Susman S. Intermediate-Range Order in Glasses and Liquids. J. Phys.: Condensed Matter 1, 1005–1008 (1989).

[b3] Murray P. G. . Ion-beam Sputtered Amorphous Silicon Films for Cryogenic Precision Measurement Systems. Phys. Rev. D. 92, 062001 (2015).

[b4] Kwo J. . Properties of High κ gate Dielectrics Gd_2_O_3_ and Y_2_O_3_ for Si. J. Appl. Phys. 89(7), 3920–3927 (2001).

[b5] Ellmer K. Past Achievements and Future Challenges in the Development of Optically Transparent Electrodes. Nat. Photon. 6, 809–817 (2012).

[b6] Greer A. L., Rutherford K. L. & Hutchings I. M. Wear Resistance of Amorphous Alloys and Related Materials. International Materials Reviews 47, 87–112 (2002).

[b7] Lee B.-S. . Investigation of the Optical and Electronic Properties of Ge_2_Sb_2_Te_5_ Phase Change Material in its Amorphous, Cubic, and Hexagonal Phases. J. Appl. Phys. 97, 093509 (2005).

[b8] Solieman A. S., Hafiz M. M., Abu-Sehly A.-h. & Alfaqeer A.-n. A. Dependence of Optical Properties on the Thickness of Amorphous Ge_30_Se_70_ Thin Films. Journal of Taibah University for Science 8, 282–288 (2014).

[b9] Berkemeier F., Abouzari M. R. S. & Schmitz G. Thickness-dependent dc Conductivity of Lithium Borate Glasses. Phys. Rev. B 76, 024205 (2007).

[b10] Davies C. E., Somekh R. E. & Evetts J. E. Magnetic Properties of Sputter-deposited Amorphous Rare Earth-transition Metal Thin Films. Vacuum 38, 797–800 (1988).

[b11] Lemoine P., Quinn J. P., Maguire P. D., Zhao J. F. & McLaughlin J. A. Intrinsic Mechanical Properties of Ultra-thin Amorphous Carbon Layers. Appl. Surf. Sci. 253, 6165–6175 (2007).

[b12] Bäumchen O., McGraw J. D., Forrest J. A. & Dalnoki-Veress K. Reduced Glass Transition Temperatures in Thin Polymer Films: Surface Effect or Artifact. Phys. Rev. Lett. 109, 055701 (2012). 23006187 10.1103/PhysRevLett.109.055701

[b13] Chu J. P. . Thin Flm Metallic Glasses: Unique Properties and Potential Applications. Thin Solid Films 520, 5097–5122 (2012).

[b14] Billinge S. J. L. & Levin I. The Problem with Determining Atomic Structure at the Nanoscale. Science 316, 561–565 (2007). 17463280 10.1126/science.1135080

[b15] Benmore C. J. A Review of High-Energy X-Ray Diffraction from Glasses and Liquids. ISRN Materials Science 19, 852905 (2012).

[b16] Cockayne D. J. H. The Study of Nanovolumes of Amorphous Materials Using Electron Scattering. Annual Review of Materials Research 159–187 (2007).

[b17] Moine P., Pelton A. R. & Sinclair R. StructuralDeterminationofSmallAmorphousVolumesbyElectronDiffraction. Journal of Non-Crystalline Solids 101, 213–222 (1988).

[b18] Eguchi T., Inoue H., Masuno A., Kita K. & Utsuno F. Oxygen Close-Packed Structure in Amorphous Indium Zinc Oxide Thin Films. Inorganic Chemistry 49, 8298–8304 (2010). 20735017 10.1021/ic1006617

[b19] Kurzman J. A., Dettelbach K. E., Martinolich A. J., Berlinguette C. P. & Neilson J. R. Structural Characteristics and Eutaxy in the Photo-Deposited Amorphous Iron Oxide Oxygen Evolution Catalyst. Chem. Mater. 27(9), 3462–3470 (2015).

[b20] Jensen K. M. Ø. . Demonstration of Thin Film Pair Distribution Function Analysis (tfPDF) for the Study of Local Structure in Amorphous and Crystalline Thin Films. IUCrJ 2, 481–489 (2015). 10.1107/S2052252515012221 PMC4547816 26306190

[b21] Fischer-Colbrie A., Bienenstock A., Fuoss P. H. & Marcus M. A. Structure and Bonding in Photodiffused Amorphous Ag-GeSe_2_ Thin Films. Phys. Rev. B 38, 12388–12403 (1988). 10.1103/physrevb.38.12388 9946180

[b22] Fuoss P. H. & Fischer-Colbrie A. Structure of a-GeSe2 from X-ray Scattering Measurements. Phys. Rev. B 38, 1875–1878 (1988). 10.1103/physrevb.38.1875 9946472

[b23] McGreevy R. L. & Howe M. A. RMC: Modeling Disordered Structures. Annual Review of Materials Science 22, 217–242 (1992).

[b24] Ding K. & Andersen H. C. Molecular-dynamics Simulation of Amorphous Germanium. Phys. Rev. B 34, 6987–6991 (1986). 10.1103/physrevb.34.6987 9939351

[b25] Lorch E. Neutron Diffraction by Germania, Silica and Radiation-damaged Silica Glasses. Journal of Physics C: Solid State Physics 2, 229–237 (1969).

[b26] Perticaroli S. . Secondary Structure and Rigidity in Model Proteins. Soft Matter 9, 9548–9556 (2013). 26029761 10.1039/c3sm50807b

[b27] Stephenson N. C. & Roth R. S. The Crystal Structure of the High Temperature Form of Ta_2_O_5_. J. Solid State Chem. 3, 145–153 (1971).

[b28] Sahu B. R. & Kleinman L. Theoretical Study of Structural and Electronic Properties of *β*−Ta_2_O_5_ and *δ*−Ta_2_O_5_. Phys. Rev. B 69, 165202 (2004).

[b29] Oehrlein G. S., d’Heurle F. M. & Reisman A. Some Properties of Crystallized Tantalum Pentoxide Thin Films on Silicon. J. Appl. Phys. 55, 3715–3725 (1984).

[b30] Maeng S., Axe L., Tyson T. & Jiang A. An Investigation of Structures of Thermal and Anodic Tantalum Oxide Films. J. Electrochem. Soc. 152, B60–B64 (2005).

[b31] Bassiri R. . Order within Disorder: The Atomic Structure of Ion-beam Sputtered Amorphous Tantala (a-Ta2O5). APL Mater. 3, 036103 (2015).

[b32] Kim N. & Stebbins J. F. Structure of Amorphous Tantalum Oxide and Titania-Doped Tantala: ^17^O NMR Results for Sol–Gel and Ion-Beam-Sputtered Materials. Chem. Mater. 23, 3460–3465 (2011).

[b33] Pradhan S. K., Mao Y., Wong S. S., Chupas P. & Petkov V. Atomic-scale Structure of Nanosized Titania and Titanate: Particles, Wires, and Tubes. Chem. Mater. 19, 6180–6186 (2007).

[b34] Winterer M. Reverse Monte Carlo Analysis of Extended X-ray Absorption Fine Structure Spectra of Monoclinic and Amorphous Zirconia. J. Appl. Phys. 88, 5635–5644 (2000).

[b35] Farrow C. L. . PDFfit2 and PDFgui: Computer Programs for Studying Nanostructure in Crystals. J. Phys.: Condensed Matter 19, 335219 (2007). 21694142 10.1088/0953-8984/19/33/335219

[b36] Bassiri R. . Probing the Atomic Structure of Amorphous Ta_2_O_5_ Coatings. Appl. Phys. Lett. 98, 031904 (2011).

[b37] Lü Xujie . Pressure-Induced Amorphization in Single-Crystal Ta_2_O_5_ Nanowires: A Kinetic Mechanism and Improved Electrical Conductivity. J. Amer. Chem. Soc. 135(37), 13947–13953 (2013). 23968372 10.1021/ja407108u

[b38] Hussain M., Niihara K. & Fukumi K. Structure Determination of Silica Tantalum Soda Glasses by X-ray Diffraction Analysis. Materials Lett. 24, 69–73 (1995).

[b39] Pickup D. M. . Structure of (Ta_2_O_5_)_x_(SiO_2_)_1 −x_ Xerogels (x = 0.05, 0.11, 0.18, 0.25 and 1.0) from FTIR, ^29^Si and ^17^O MAS NMR and EXAFS. J. Mater. Chem. 10, 1887–1894 (2000).

[b40] FitzGerald V., Pickup D. M., Drake K. O., Smith M. E. & Newport R. J. A High Energy X-ray Diffraction Study of Sol-gel Derived (Ta_2_O_5_)_x_(SiO_2_)_1–x_ Glasses (x = 0.05, 0.11 and 0.25)-Elucidating the Role of Tanalum in Silica. Journal of Sol-Gel Science and Technology 44, 153–159 (2007).

[b41] Wu Y.-N., Li L. & Cheng H.-P. First-principles Studies of Ta_2_O_5_ Polymorphs. Phys. Rev. B 83, 144105 (2011).

[b42] Bassiri R. . Investigating the Medium Range Order in Amorphous Ta_2_O_5_ Coatings. J. Phys.: Conference Series 522, 012043 (2014).

[b43] Hart M. J. . Medium Range Structural Order in Amorphous Tantala Spatially Resolved with Changes to Atomic Structure by Thermal Annealing. Journal of Non-Crystalline solids 438, 10–17 (2016).

[b44] Lechner P. . Silicon Drift Detectors for High Resolution Room Temperature X-ray Spectroscopy. Nuclear Instruments and Methods in Physics Research Section A: Accelerators, Spectrometers, Detectors and Associated Equipment 377, 346–351 (1996).

[b45] Mei Q., Benmore C. J. & Weber J. K. R. Structure of Liquid SiO_2_: A Measurement by High-Energy X-Ray Diffraction. Phys. Rev. Lett. 98, 057802 (2007). 17358901 10.1103/PhysRevLett.98.057802

[b46] Elliott S. R. Origin of the First Sharp Diffraction Peak in the Structure Factor of Covalent Glasses. Phys.Rev. Lett. 67, 711–714 (1991). 10044969 10.1103/PhysRevLett.67.711

[b47] Petkov V. . Structure-Properties Correlation in Si Nanoparticles by Total Scattering and Computer Simulations. Chem. Mater. 25(11), 2365–2371 (2013).

[b48] Yamakawa N., Jiang M., Key B. & Grey C. P. Identifying the Local Structures Formed During Lithiation of the Conversion Material, Iron Fluoride, in a Li Ion Battery: a Solid-State NMR, X-ray Diffraction, and Pair Distribution Function Analysis Study. J. Amer. Chem. Soc. 131, 10525–10536 (2009). 19585988 10.1021/ja902639w

[b49] Dambournet D. . Dual Lithium Insertion and Conversion Mechanisms in a Titanium-based Mixed-anion Nanocomposite. J. Amer. Chem. Soc 133, 13240–13243 (2011). 21809881 10.1021/ja204284h

[b50] Petkov V., Qadir D. & Shastri S. D. Rapid Structure Determination of Disordered Materials: Study of GeSe_2_ glass. Solid State Comm. 129, 239–243 (2004).

