# Supplementary material for: Measurement and Modeling of Short and Medium Range Order in Amorphous Ta2O5 Thin Films
Source: Sci Rep. 2016 Aug 26;6:32170. doi: 10.1038/srep32170 (PMC4999889; doi:10.1038/srep32170)
Supplement: Supplementary Information [file srep32170-s1.pdf]

# Supplementary Information: Measurement and Modeling of Short and Medium Range Order in Amorphous $Ta_2O_5$ Thin Films

*Badri Shyam[1], Kevin H. Stone[1], Riccardo Bassiri[2], Martin M. Fejer[2], Michael F. Toney[1] and Apurva Mehta[1]**

[1]Stanford Synchrotron Radiation Lightsource, SLAC National Accelerator Laboratory, Menlo Park CA 94025, United States

[2]Department of Applied Physics, Stanford University, Stanford CA 94305, United States

e-mail address: mehta@slac.stanford.edu

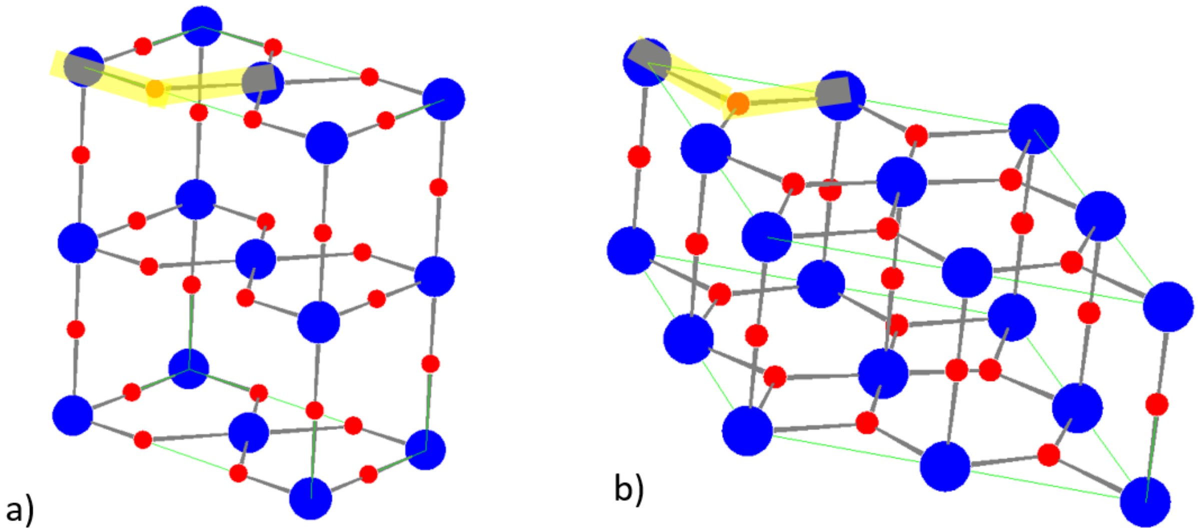


**Figure 1**: Schematic structure of a) β–Tantala, as reported by Aleshina and Loginova[1] b) δ–Tantala as calculated by Fukumoto and Miwa.[2] The blue spheres indicate Ta ions and the red sphere indicate O ions. The kinked Ta-O-Ta PSU is high-lighted in yellow.

## References

1. Aleshina, L. A., & Loginova, S. V. Rietveld analysis of X-ray diffraction pattern from β-$Ta_2O_5$ oxide. *Crystallogr. Rep.*, **47,** 415-419 (2002).

2. Fukumoto, A., & Miwa, K. Prediction of hexagonal $Ta_2O_5$ structure by first-principles calculations. *Phys. Rev.*, B **55**, 11155-11160 (1997).
